# Hypocalcemia and a Positive Metabolic Screen for Severe Combined Immunodeficiency in an 11-Day-Old Male With DiGeorge Syndrome

**DOI:** 10.7759/cureus.10625

**Published:** 2020-09-23

**Authors:** Jessica Ford, Jose M Pena, Shane C Rainey

**Affiliations:** 1 Pediatrics, University of Illinois College of Medicine at Peoria, Peoria, USA; 2 Child Health, University of Arizona College of Medicine - Phoenix, Phoenix, USA

**Keywords:** digeorge syndrome, genetics, endocrinology, hypocalcemia, pediatrics

## Abstract

22q11 deletion syndrome (22q11DS), also known as DiGeorge syndrome or velocardiofacial syndrome, is the most common human genetic microdeletion. Hypocalcemia secondary to hypoparathyroidism is a common finding in this condition and may present with seizures. We describe a case of an 11-day-old male presenting with hypocalcemic seizures and a positive newborn screen for severe combined immunodeficiency as the primary manifestations of 22q11DS. Given the potential for wide phenotypic variability, clinicians should maintain a high index of suspicion for this syndrome, especially in the neonate presenting with hypocalcemia.

## Introduction

22q11 deletion syndrome (22q11DS) is the most common genetic microdeletion syndrome encountered in humans and may have wide phenotypic variability [1]. The clinical manifestations commonly include cardiac defects, facial dysmorphisms, cleft palate, thymic hypoplasia, and hypocalcemia, although the presentation may vary widely between individual patients [2]. We describe here an unusual case of a neonate presenting with seizures and a noted positive newborn screen (NBS) for severe combined immunodeficiency syndrome (SCID) as the first signs of his underlying genetic deletion. Given the variability in clinical presentation, clinicians should maintain a high index of suspicion for 22q11DS in the right clinical context.

## Case presentation

An 11-day-old male presented to the emergency department (ED) with intermittent twitching episodes. He was born at 38 and 1/7 weeks gestation via cesarean section for fetal distress to a Group B *Streptococcus *and serology negative (hepatitis B, syphilis, human immunodeficiency virus, and rubella immune) mother who abused opiates, benzodiazepines, and cocaine perinatally. Other than one episode of hypoglycemia that corrected with feeding, his nursery stay was unremarkable. He was feeding well, gaining weight appropriately, and was discharged on day of life three. He was seen by his primary care physician on day of life five for an uncomplicated well-newborn evaluation. Five days prior to presentation, on day of life six, he started to have clusters of bilateral upper and lower extremity jerking two to three times daily, each lasting less than a minute. On the day of presentation, he had two episodes that occurred within five minutes, prompting their presentation in the ED. He had no fevers or other symptoms and was not taking any medications. There was no family history of seizures.

On physical examination, he was afebrile with a weight of 3.07 kg (eighth percentile) and a head circumference of 33 cm (12th percentile). He had a right-sided facial droop (noted at the corner of his mouth with a shallow right nasolabial fold) and small, low-set auricles bilaterally. There was no cleft lip, cleft palate, hypertelorism, or micrognathia. Cardiac auscultation revealed a II/VI systolic ejection murmur at the left upper sternal border. Other than the right-sided facial droop, his neurological exam was unremarkable with good tone, a strong suckle reflex, and symmetric Moro, palmar, and plantar grasp reflexes. The remainder of his examination findings was normal.

**Figure 1:**
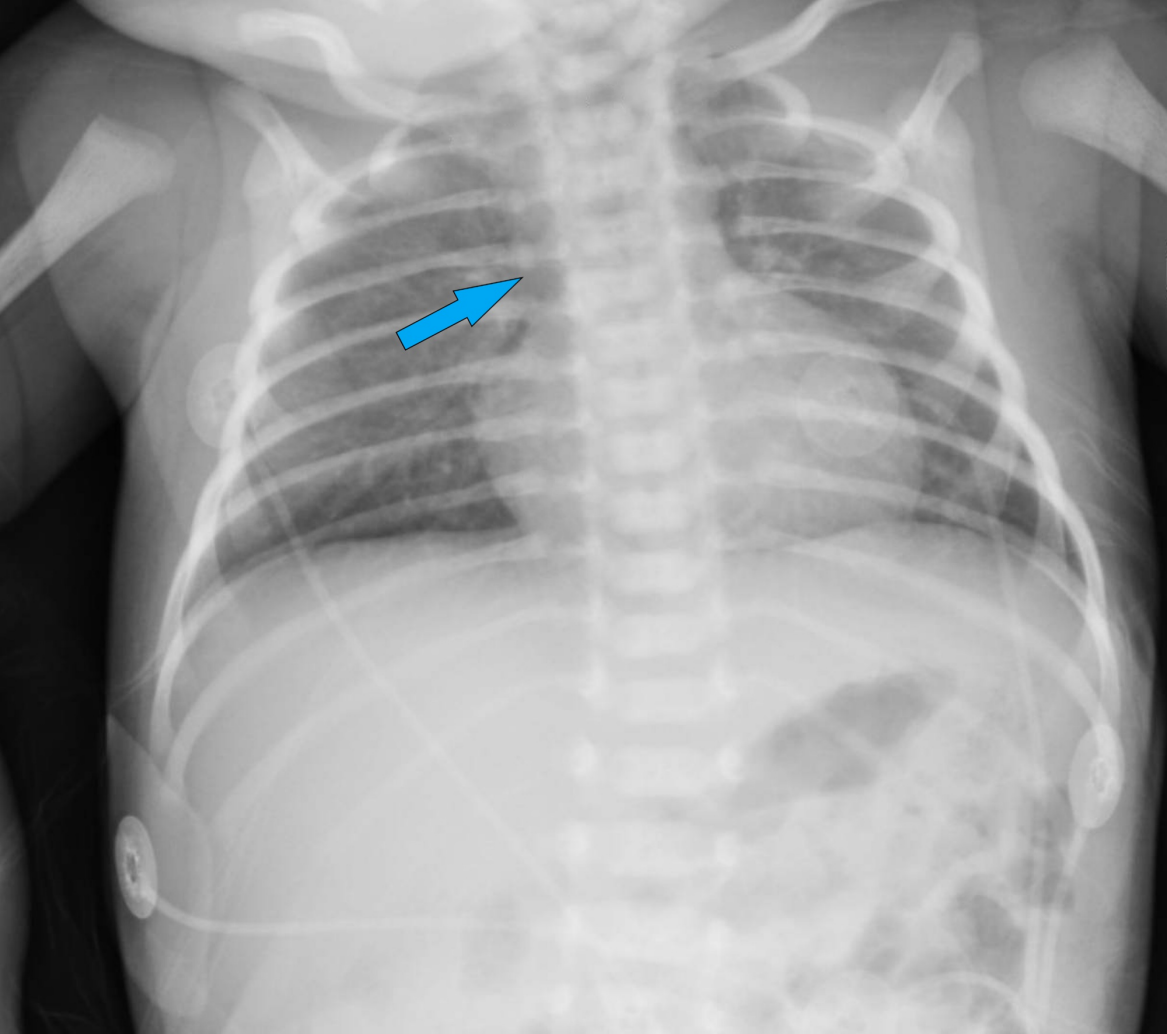
Chest X-ray demonstrating absence of the thymic shadow (arrow).

Initial laboratory evaluation revealed a calcium level of 6.9 mg/dL, a phosphorus level of 9.4 mg/dL, a parathyroid hormone (PTH) level of 11 pg/mL (reference range 13-85 pg/mL), and a vitamin D level of 12 ng/mL. A urine drug screen was negative. A chest x-ray was performed and demonstrated an absent thymic shadow (Figure 1).

**Figure 2:**
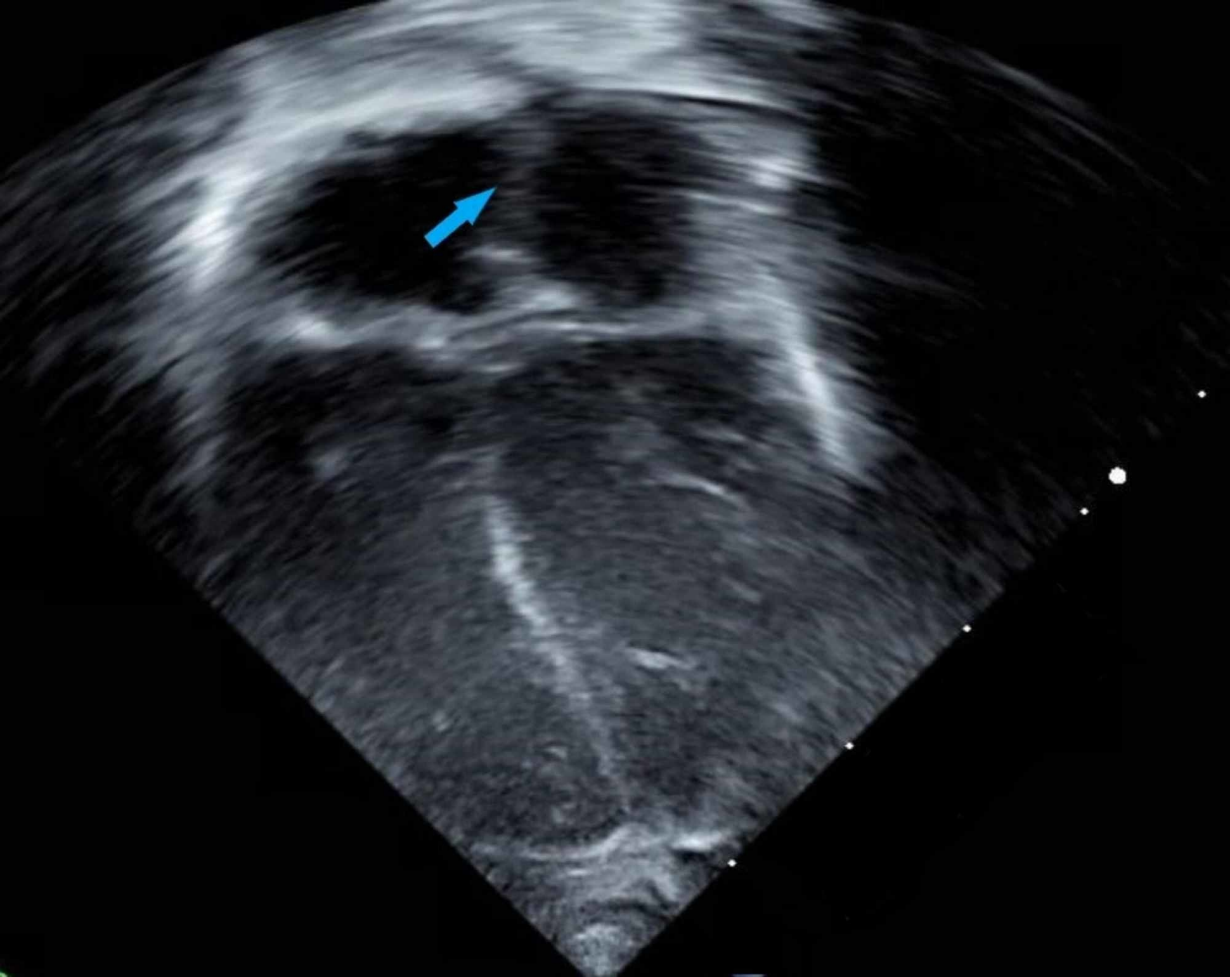
Echocardiogram apical four-chamber view showing a patent foramen ovale (arrow).

**Figure 3:**
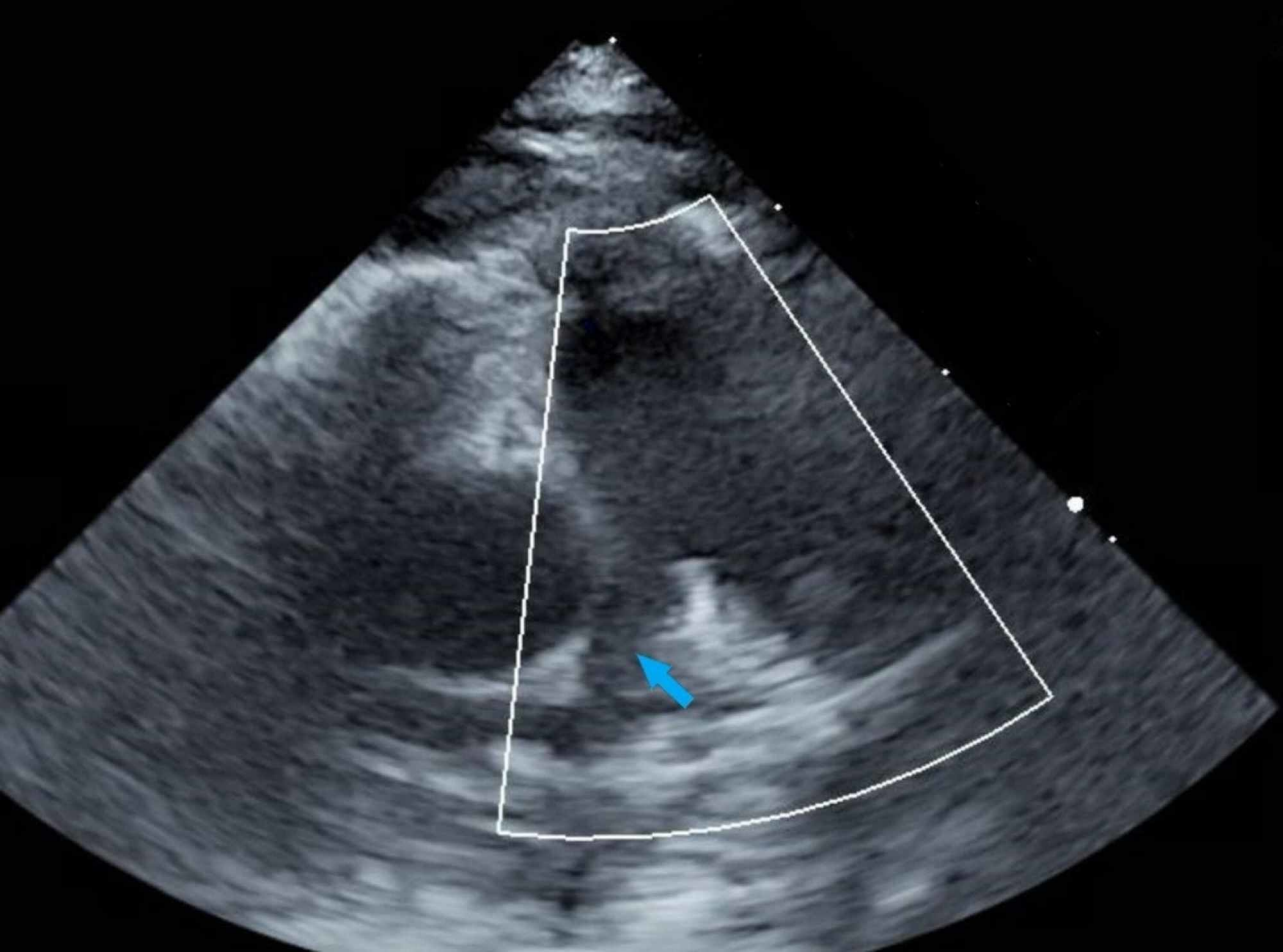
Echocardiogram parasternal short axis view showing peripheral pulmonary artery stenosis (arrow).

The infant was admitted to the intensive care unit and treated with a single dose of 100 mg/kg of intravenous calcium gluconate with resolution of his tetany. The results of a complete blood count, the remainder of his comprehensive metabolic profile, urinalysis, and urine and blood cultures were negative. Endocrinology was consulted and started the patient on 250 mcg/day of calcitriol and calcium carbonate at 75 mg/kg/day of elemental calcium for primary hypoparathyroidism. He was also started on 2000 IU/day of ergocalciferol for vitamin D deficiency. After initiating treatment, he remained symptom-free throughout his week of admission. An echocardiogram showed peripheral pulmonary artery stenosis and a patent foramen ovale with otherwise normal cardiac anatomy (Figures 2, 3).

On admission day two, the state NBS returned positive for SCID, prompting further evaluation with T and B cell populations and a chromosomal microarray to evaluate for 22q11DS. A repeat newborn screen was sent at that time and also returned positive for SCID, and he was referred to immunology. T and B cell population testing returned with a suppressed but non-diagnostic absolute T-cell count of 1603/mm3 (reference range 2500-5500/mm3). Immunoglobulin levels and total B-cell count were within normal limits. His chromosomal microarray demonstrated a 2.5 megabase deletion at the 22q11.2 gene locus, confirming the diagnosis of 22q11DS, also known as DiGeorge syndrome. The patient's medications were carefully adjusted during admission, and he was discharged home to continue ergocalciferol, calcitriol, and calcium carbonate with close follow-up with endocrinology, genetics, immunology, and his primary care physician. He has continued to be clinically well post-discharge with stable labs and no further seizure-like activity or tetany. 

## Discussion

Hypocalcemia is a common problem in neonates, although many infants are asymptomatic and often only identified incidentally during laboratory testing. Among symptomatic infants, a majority present with irritability, muscle twitching, and focal to generalized tonic-clonic seizures [3,4]. The differential diagnosis of neonatal hypocalcemia can be divided into early and late onset depending on whether the condition develops before or after 72 hours of life. Early hypocalcemia is thought to be a worsening of the physiologic decline in serum calcium levels after birth and is most commonly seen in premature, very low birth weight, and growth-restricted infants [5]. Furthermore, about 20 percent of infants born to diabetic mothers will experience hypocalcemia [6]. Other causes of early hypocalcemia include birth asphyxia, maternal hyperparathyroidism, hypomagnesemia, and hypoparathyroidism, either due to defective release of PTH, hypoplastic, or absent glands as seen in 22q11DS [7]. Late hypocalcemia is much more likely to have a symptomatic presentation and is most frequently seen in infants fed with cow’s milk-based formulas that are high in phosphorus [8]. Critically ill infants may be at risk for hypocalcemia if they receive sodium bicarbonate infusions, blood transfusions, or lipid containing total parenteral nutrition, each of which can favor development of calcium complexes, thereby decreasing the ionized calcium concentration. Vitamin D deficiency is also a noted cause of late hypocalcemia, although the vitamin D level at which hypocalcemia occurs in infancy is not well defined [4,9].

22q11DS, also known as DiGeorge Syndrome or velocardiofacial syndrome, is the most common microdeletion syndrome reported in humans, occurring in approximately one in 3,000 newborns [1]. The clinical presentation can be remembered by the mnemonic “CATCH 22,” consisting of cardiac defects, abnormal facies, thymic hypoplasia, cleft palate, hypocalcemia, and a deletion on chromosome 22 [2]. Conotruncal defects are the most commonly noted congenital heart abnormalities and usually present in the neonatal period. Hypocalcemia is typically early onset and is amenable to treatment with calcium and vitamin D [7,10]. Given that parathyroid hypoplasia typically results in early hypocalcemia, our patient's late presentation with significant seizure activity was unusual.

Immunodeficiency is identified in up to 75% of individuals with 22q11DS and can affect both cell-mediated and humoral elements of the immune system [11]. Thymic hypoplasia typically suppresses the normal development of T-cell lymphocyte populations, as seen in our patient. However, suppression of these lymphocyte populations in patients with 22q11DS to a degree that would generate a positive NBS for SCID is less frequent, occurring in 0.01%-3% of cases [11-14]. 

A variety of facial dysmorphisms are characteristic of 22q11DS and include a long face; hypertelorism; low nasal bridge; micrognathia; asymmetric facial movements; and small, low-set ears, although there is wide phenotypic variability. The diagnosis is made via genetic testing, specifically with fluorescence in situ hybridization targeted to the suspected gene region or chromosomal microarray [1]. Management is individualized to the patient based on their phenotypic manifestation and is multidisciplinary involving specialists from genetics, endocrinology, immunology, otolaryngology, plastic surgery, and speech therapy.

Overall, the clinical findings of tetany, low-set auricles, facial droop, hypocalcemia, and a positive newborn screen for SCID led us to consider 22q11DS in our patient, which was confirmed by genetic testing. The late-onset hypocalcemia, absence of orofacial cleft defects, more severe congenital heart disease, and relatively few facial dysmorphisms in our patient may be different than other presentations of this genetic disease. Given the wide phenotypical variability in clinical presentation, a high index of suspicion is necessary for the diagnosis of 22q11DS.

## Conclusions

22q11DS is the most common microdeletion in humans and should remain on the differential diagnosis of neonates presenting with hypocalcemia. Given the potential for phenotypic variability, clinicians should maintain a high index of suspicion for the syndrome in the correct clinical context, including a neonate presenting with seizures or a positive NBS for SCID.

## References

[1] McDonald-McGinn DM, Sullivan KE, Marino B (2015). 22q11 deletion syndrome. Nat Rev Dis Primers.

[2] Wilson DI, Burn J, Scambler P, Goodship J (1993). DiGeorge syndrome: part of CATCH 22. J Med Genet.

[3] Habel A, Herriot R, Kumararatne D (2014). Towards a safety net for management of 22q11.2 deletion syndrome: guidelines for our times. Eur J Pediatr.

[4] Thomas TC, Smith JM, White PC, Adhikari S (2012). Transient neonatal hypocalcemia: presentation and outcomes. Pediatrics.

[5] Hsu SC, Levine MA (2004). Perinatal calcium metabolism: physiology and pathophysiology. Semin Neonatol.

[6] Rosenn B, Miodovnik M, Tsang R (1996). Common clinical manifestations of maternal diabetes in newborn infants: implications for the practicing pediatrician. Pediatr Ann.

[7] Cheung ENM, George SR, Costain GA, Andrade DM, Chow EWC, Silversides CK, Bassett AS (2014). Prevalence of hypocalcemia and its associated features in 22q11.2 deletion. Clin Endocrinol (Oxf).

[8] Specker BL, Tsang RC, Ho ML, Landi TM, Gratton TL (1991). Low serum calcium and high parathyroid hormone levels in neonates fed ‘humanized’ cow’s milk-based formula. Am J Dis Child.

[9] Yilmaz B, Aygun C, Cetinoglu E (2018). Vitamin D levels in newborns and association with neonatal hypocalcemia. J Matern Fetal Neonatal Med.

[10] Kobrynski LJ, Sullivan KE (2007). Velocardiofacial syndrome, DiGeorge syndrome: the chromosome 22q11.2 deletion syndromes. Lancet.

[11] Barry JC, Crowley TB, Jyonouchi S, Heimall J, Zachai EH, Sullivan KE, McDonald-McGinn DM (2017). Identification of 22q11.2 deletion syndrome via newborn screening for severe combined immunodeficiency. J Clin Immunol.

[12] Kwan A, Church JA, Cowan MK (2013). Newborn screening for SCID and T cell lymphopenia in California: results of the first two years. J Allergy Clin Immunol.

[13] Routes JM, Grossman WJ, Verbsky J, Laessig RH, Hoffman GL, Brokopp CD, Baker MW (2009). Statewide newborn screening for severe T-cell lymphopenia. JAMA.

[14] Vogel BH, Bonagura V, Weinberg GA (2015). Newborn screening for SCID in New York state: experience from the first two years. J Clin Immunol.

